# Toxicity and anthelmintic efficacy of chitosan encapsulated bromelain against gastrointestinal strongyles in Small East African goats in Kenya

**DOI:** 10.14202/vetworld.2020.177-183

**Published:** 2020-01-25

**Authors:** Shukuru Wasso, Naomi Maina, John Kagira

**Affiliations:** 1 Department of Molecular Biology and Biotechnology, Pan African University, Institute of Basic Sciences, Technology and Innovation, P.O. Box 62000-00200, Nairobi, Kenya; 2 Department of Biochemistry, Jomo Kenyatta University of Agriculture and Technology, P.O. Box 62000-00200, Nairobi, Kenya; 3 Department of Animal Sciences, Jomo Kenyatta University of Agriculture and Technology, P.O. Box 62000-00200, Nairobi, Kenya

**Keywords:** anthelmintic efficacy, bromelain, chitosan, goats, nanoencapsulation, toxicity

## Abstract

**Background and Aim:**

The development of resistance to anthelmintic drugs has prompted research into alternative methods of controlling intestinal nematodes in ruminants. This study aimed at evaluating the *in vitro* and *in vivo* anthelmintic efficacy and toxicity of chitosan encapsulated bromelain in Small East African goats in Kenya.

**Materials and Methods:**

Adult mortality assay was performed using live *Haemonchus contortus* worms treated with encapsulated bromelain solution ranging from 0.125 mg/ml to 2 mg/ml. Percentage mortality of worms was calculated after 24 h and the lethal concentration 50% (LC_50_) determined. For the *in vivo* study, 18 healthy male indigenous goats were divided into six groups of three goats each. The encapsulated bromelain was orally administered in increasing dosages (3-30 mg kg) once daily, for 14 days. The packed cell volume (PCV), aspartate aminotransferase (AST), alanine aminotransferase (ALT), urea, creatinine, and fecal egg count (FEC) were determined on a weekly basis. At the end of the study, the goats were sacrificed and gross pathology and histopathology of main organs assessed.

**Results:**

Albendazole had the highest (p<0.05) anthelmintic effect on the worms. An LC_50_ of 0.05 mg/ml, 0.445 mg/ml, and 0.155 mg/ml was observed for albendazole, plain bromelain, and encapsulated bromelain, respectively. The PCV of treated and untreated goats did not show any significant difference (p>0.05), varied from 29.3% to 35.1%, and was within the normal range of the animal. Likewise, no significant differences (p>0.05) were observed between the AST, ALT, urea, and creatinine levels of treated and the control (non-treated) goats. No adverse clinical symptoms, toxicity of the main organs, and mortality in goats were associated with the chitosan encapsulated bromelain after administration of dose up to 30 mg/kg for 14 days. Therefore, the lethal dose 50 of encapsulated bromelain may be considered to be >30 mg/kg. On day 28 post-treatment, the encapsulated bromelain showed a higher *in vivo* FEC reduction (68.8%) as compared to the plain bromelain (32.4%).

**Conclusion:**

Our results show that bromelain encapsulated in chitosan may be safe and effective in reducing the burden of gastrointestinal tract strongyle nematodes in goats. However, there is a need for further studies to establish the dosage of the encapsulated bromelain to be administered in a single dose for the treatment of goats against gastrointestinal strongyles. In addition, species-specific studies on the efficacy of encapsulated bromelain on strongyles are necessary to evaluate its effectiveness against the entire Strongyloididae family.

## Introduction

Helminthosis is an important disease associated with the infection of helminths in the gastrointestinal tract of ruminants [1]. Currently, it is one of the most economically important diseases of livestock in tropics and has a significant economic impact on the well-being of farmers in developing countries [2]. The disease causes the suppression of weight gain, reduction of reproductive performances and leads to high mortality in extreme cases [3,4]. Farmers rely on the aggressive use of anthelmintics to treat and control helminthosis. While commercial anthelmintic usage has shown beneficial effects on animal health, it is also associated with problems, including consumer concern over potential synthetic drug residues in animal products [5,6], and loss of efficacy as a result of the emergence of resistance [7,8]. Given the widespread occurrence of resistance, there is a need for new alternatives involving the development of new drugs with different modes of action, with a specific focus on products arising from plants [9].

Pineapple fruits, stem, and peels are good sources of bromelain which have been proven to have anthelmintic activities [10]. The use of bromelain as anthelmintics has faced some constraints including administration challenges [11]. Studies have shown that after administration, bromelain anthelmintic activity is lowered by the low pH found in the abomasum of ruminants. Moreover, the rumen microbiota can cause deterioration of the drug efficacy, resulting in an ineffective contact between worms and drugs [11]. The study by Hunduza [12] showed that the encapsulation of bromelain with chitosan enhanced it’s *in vitro* activity against all the stages of *Haemonchus contortus* isolated from goats. It revealed that encapsulated bromelain had a higher (p>0.05) egg hatch inhibition activity (half maximal inhibitory concentration [IC_50_=0.249]) than extracted (IC_50_=0.325) and pure bromelain (IC_50_=0.327). The study also indicated that encapsulated bromelain had significantly higher (p<0.05) absorbance than that of plain bromelain at low pH (2.0 and 3.0) showing that chitosan nanocarriers stabilize and maintain the activity of bromelain. This implies that bromelain encapsulated in chitosan can be used to target and affect parasites found in the abomasum of ruminants which has a low pH. However, there is a need to further conduct research on the drug by evaluating its toxicity and efficacy against helminthosis *in vivo* in goats.

Therefore, this study aimed at evaluating the *in vitro* and *in vivo* efficacy and toxicity of a bromelain chitosan formulation in Small East African goats in Kenya.

## Materials and Methods

### Ethical approval

Approval for animal experiments was obtained from the Jomo Kenyatta University of Agriculture and Technology (JKUAT) Animal Ethics Committee (REF: JKU/2/4/896B). The protocols were approved by the Institutional Animal Care and Use Committee at JKUAT and conducted in compliance with Kenya’s National ethical standards to minimize animal suffering.

### Study site

The study was carried out at JKUAT, Kiambu County, Kenya. The university is located at latitude 1°05 S and longitude 37°00 E. It lies at an altitude of 1525 *m* above sea level and receives an annual rainfall of 850 *mm* with temperatures ranging from 13°C to 26°C [13].

### Study animals

Eighteen Small East African healthy male indigenous goats, aged between 8 and 30 months old and weighing between 13 and 21 kg, were purchased from local farmers. They were ear-tagged and kept in a goat house where they were acclimatized to the diet over the course of 14 days before commencing the study. Animals were group-housed in pens of size 2 m×2 m (three goats in each) inside the main goat house which was located within the JKUAT. Each animal was screened for the presence of strongyle eggs by fecal egg count (FEC) examination before the start of the experiment. Goats were fed on 1.5 kg of concentrate feed and 1 kg of wheat hay twice each day (at 9 a.m. and 3 p.m.). The concentrate feed comprised beet liquid molasses, maize germ, and soybean meal (Aroma Feed Suppliers, Kenya). Supplementation of essential minerals was done using feed blocks (Aroma Feed Suppliers, Kenya). Along with the experiment, albendazole (Sigma-Aldrich, USA) was administered to goats as a positive control. Commercial bromelain (Sigma-Aldrich, USA) was used as a parallel control test.

### Extraction of bromelain and encapsulation in chitosan nanoparticles

Bromelain extraction was performed as described by Kahiro *et al*. [14]. The extracted bromelain was purified using a 10kDa dialysis membrane. The ionic gelation method was used to encapsulate bromelain into chitosan [12] where equal volume (30 ml) of extracted bromelain (4 mg/ml) was mixed with 1% sodium tripolyphosphate (STPP) and rotary mixed for 1 min. Using a syringe, 12 ml of the bromelain-STPP mixture was added to 20 ml of 1% chitosan under vigorous and continuous stirring. The resultant suspension was centrifuged at 15,000 rpm for 45 min and the obtained pellet washed with distilled water before freeze-drying. The aliquots of bromelain loaded chitosan nanocarrier pellet were frozen at −60°C and placed in the freeze-dryer (MRC equipment manufacturer, Israel). The freeze-drying was allowed to run until all the samples were completely dried. Successful conjugation of bromelain to the chitosan nanoparticles was confirmed by Fourier transform infrared spectrophotometer analysis.

### *In vitro* adult worm mortality assay

Adult mortality assay was conducted according to the procedure by Eguale *et al*. [15] and Hunduza [12]. Briefly, ten actively moving worms were placed in Petri dishes and exposed to encapsulated bromelain solution ranging from 0.125 mg/ml to 2 mg/ml. Albendazole prepared in concentrations ranging from 0.125 mg/ml to 2 mg/ml was used as the positive control while phosphate-buffered saline was used as the negative control. Commercial bromelain was also prepared in concentrations ranging from 0.125 mg/ml to 2 mg/ml and was used as a parallel control test. Each test was done in triplicate. After 24 h, the number of live and dead worms was counted and the percentage mortality calculated using the formula:


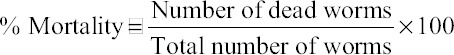


### Animal treatments

Treatment groups were formed after randomization based on the number of eggs per gram (EPG) of feces (EPG values), such that the mean EPG of the animals in each group was more than 500 [16]. Each group had three animals. The treatment was done orally every morning (9 a.m.) for 14 days. Groups 1, 2, and 3 received 30 mg/kg, 10 mg/kg, and 3 mg/kg of encapsulated bromelain, respectively. Group 4 received 30 mg/kg of plain bromelain. Group 5 was the positive control (Albendazole, 7.5 mg/kg body weight) and Group 6 was the negative infected non-treated control. The above dosages were chosen following the World Association for the Advancement of Veterinary Parasitology guidelines for dose determination [17] and the results of the bromelain toxicity tests obtained in the previous studies [18-20].

### *In vivo* toxicity assessment

#### Clinical observations

Goats were fasted overnight before dosing. Following the period of fasting, the animals were weighed and then the test substance was administered orally using drenching guns. Observations were made and recorded systematically and continuously as per the guidelines [21]. Animals were observed individually during the entire study period. Special attention was given during the first 4 h and daily thereafter, for a total of 14 days to observe any death or changes in general behavior and other physiological activities. Observations included changes in skin and fur, eyes and mucous membranes, respiratory system, and behavior pattern. Attention was also directed to observations of salivation, diarrhea, lethargy, and sleep in the animals [21].

Temperature and bodyweight of goats were measured at 09:00 am using a digital thermometer (Kruuse Digital Thermometer; Jørgen Kruuse) and a 100 kg spring balance scale (Salter Model, Capital Scales – Pretoria, South Africa), respectively. This was done before treatment and once a week during the experiment period. Changes in the weight of individual goats were calculated and compared with that of the control animals. Changes were considered as a result of the adverse effects of drugs if the body weight loss observed was more than 10% of the initial recorded body weight [22].

#### Sample collection

Blood samples (3 ml) from each goat were drawn from the jugular vein in ethylenediaminetetraacetic acid test tubes using 3 ml syringes with 3/4-inch, 20-gauge needles. This was done at 09:00 am weekly.

#### Packed cell volume (PCV)

The PCV was determined using the microhematocrit method [23,24]. Briefly, an aliquot of blood with anticoagulant from each goat was put in micro-capillary tubes and then centrifuged at 14,000 rpm for 10 min. After centrifugation, samples were analyzed for PCV using a micro-capillary reader (Hawksley, England).

#### Determination of serum biochemical parameters

Aspartate aminotransferases (AST), alanine aminotransferases (ALT), urea, and creatinine were analyzed using standard diagnostic test kits on automated clinical biochemistry analyzer (Reflotron Plus System^®^, model: Cobas 4800 Detection Analyzer; Mumbai, India).

#### Gross pathology and histopathology

At the end of the experimental period, the goats were sacrificed and examined for gross pathology. Parts of the following organs were preserved in 10% buffered formalin: Liver, kidney, spleen, and heart. Thereafter, the organs were processed for histopathology and the prepared slides examined under the microscope using the procedure as described by Slaoui and Fiette [25].

### Assessment of the *in vivo* anthelmintic efficacy

Fecal samples were collected once a week, for 4 weeks, from the rectum of the goats using fresh gloves. Aliquots of 2 g of the fecal sample from each goat were placed in a plastic bottle (Indosurgicals Pvt. Ltd., New Delhi, India) for analysis.

The fecal samples were analyzed using a modified McMaster technique [26] with a precision of 100 EPG of feces using an Olympus B 201 microscope (Optical Element Corporation, Melville, USA) at 10×. FEC reduction (FECR) percentage was calculated using the formula:

% FECR=100×[1−(T_2_/T_1_)]

T_1_=Mean pre-treatment FEC in treatment group

T_2_=Mean post-treatment FEC in treatment group [27].

### Statistical analysis

All statistical analyses and graphical presentations were carried out using R (version 3.6.0) (Foundation for Statistical Computing, Vienna, Austria) and GraphPad (Version 7.02) (GraphPad Software, Inc., La Jolla, CA, USA), respectively. Tests of significance for different drug concentrations on worm mortality were conducted by analysis of variance (ANOVA) and the significance level was set at p<0.05. Regression analysis between the log concentrations and probit-transformed responses was used to determine the lethal concentration 50% (LC_50_). Pre- and post-treatment comparisons of FEC, PCV, weight, and temperature were done using ANOVA (p<0.05).

## Results

### *In vitro* worm mortality assay

**Figure-1:**
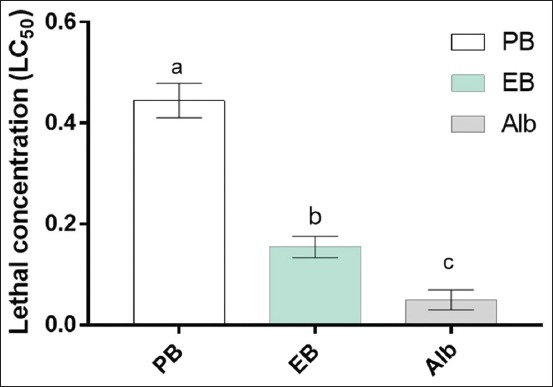
Mean lethal concentration 50% of bromelain, encapsulated bromelain, and albendazole. Data represent means with standard deviations of three replicates analyzed using R version 3.6.0. Small letters on top of each bar compare means, according to Tukey’s honestly significant difference test (p≤0.05). Different letters indicate significantly different values. PB: Plain bromelain, EB: Encapsulated bromelain, Alb: Albendazole.

At all concentrations, albendazole had the greatest effect on the worms with 1 mg/ml achieving 100% worm mortality. Encapsulated bromelain showed higher activity compared to the plain bromelain and achieved 100% worm mortality at 1 mg/ml. Albendazole had significantly (p<0.05) higher activity than encapsulated bromelain and plain bromelain. The LC_50_ observed were 0.05 mg/ml, 0.155 mg/ml, and 0.445 mg/ml for albendazole, encapsulated bromelain, and plain bromelain, respectively (Figure-1).

### Encapsulated bromelain toxicity assessment

#### Clinical observations

Administration of dose up to 30 mg/kg encapsulated bromelain did not reveal any mortality in goats during the entire observation period. Therefore, lethal dose 50 (LD_50)_ of encapsulated bromelain may be considered to be >30 mg/kg. No treatment-related clinical symptoms of toxicity were observed during the experimental period at any of the three doses of encapsulated bromelain, as well as for the plain bromelain (30 mg/kg) and albendazole (7.5 mg/kg) used as parallel controls. The skin, fur, mucous membrane, urination, water intake, and food intake of goats were found to be normal before and after treatment. Lethargy, diarrhea, inactivity, rapid breathing, excessive salivation, liquid secretion from eyes, and rapid breathing were not observed. Goat body temperatures varied from 38.1°C to 39.2°C, which is within the normal range of the animal [28]. No significant variation of temperature and body weight (p<0.05) was observed between the control non-treated goats and the encapsulated bromelain treated goats.

#### Necropsy macroscopic observation and histological examination

**Figure-2:**
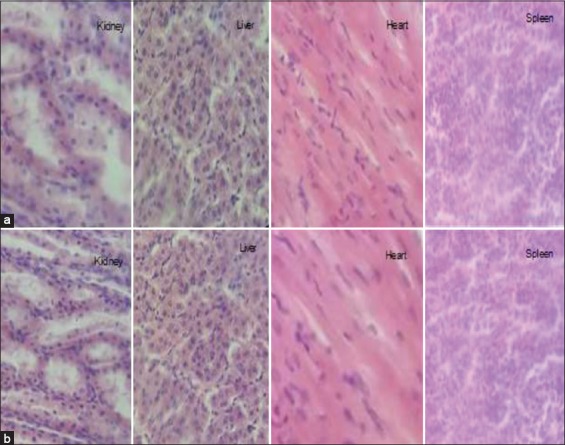
Histological examinations of the kidney, liver, heart, and spleen of goats after 14 days of repeated-dose toxicity study. Representative photomicrographs from kidney, liver, heart, and spleen sections stained with hematoxylin and eosin (H&E), respective groups: (a) Control non-treated group, (b) encapsulated bromelain treated group (30 mg/kg), H&E 400×.

No treatment-related pathological changes of internal organs were observed in necropsy examination. Hematoxylin and eosin staining of liver showed normal hepatic architecture, hepatocytes, and hepatic sinusoids. Histological assay of the kidney from encapsulated bromelain treated groups showed normal renal architecture with a normal appearance of glomerulus and tubules. The spleen appeared normal with no histopathological alteration. Heart sections of encapsulated bromelain treated groups presented a normal myocardial architecture (Figure-2).

### Effect of the plant extract on the PCV and serum biochemical parameters

The PCV of untreated goats varied between 29.3% and 35.1%. The mean PCV of treated goats was similar to that of the control untreated group (p>0.05). ALT and AST levels of untreated goats ranged from 14.07 to 16.17 U/L and 105.6 to 115.5 U/L, respectively, which were within the normal range [29]. No significant difference (p>0.05) was observed between the AST and ALT levels of treated and the control non-treated goats. Likewise, urea and creatinine levels of untreated goats varied from 5.6 to 6.3 mmol/L and 55.4 to 63.07 µmol/L, respectively, and were within the normal range of the animal [29]. There was no significant difference between the urea and creatinine levels of treated goats and the control non-treated goats.

### *In vivo* anthelmintic efficacy assessment

**Figure-3:**
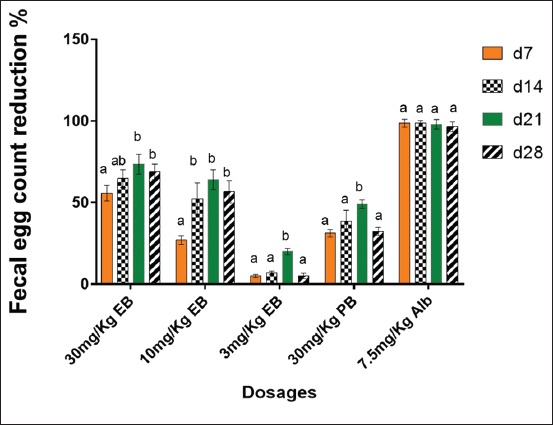
Fecal egg count reduction test. Positive values indicate a relative reduction of the number of EPG of feces and negative values a relative increase. Data represent means with standard deviations of three replicates analyzed using R version 3.6.0. Small letters on top of each bar compare means at different days post-treatment according to Turkey’s honestly significant difference test (p≤0.05). Different letters indicate significantly different values. FECR: Fecal egg count reduction, PB: Plain bromelain, EB: Encapsulated bromelain, Alb: Albendazole, EPG: Number of eggs per gram of feces, d7: Day 7 post-treatment, d14: Day 14 post-treatment, d21: Day 21 post-treatment, d28: Day 28 post-treatment.

The percentage reduction in strongyle eggs was significantly higher (p<0.05) for the goats treated with 7.5 mg/kg of albendazole than those treated with encapsulated bromelain at any of the three tested doses (30, 10, and 3 mg/kg). Similarly, a significant (p<0.05) gradual increase in percentage reduction of strongyles eggs was observed over time for goats treated with the plain and encapsulated bromelain. On 28^th^ day post-treatment, 68.8%, 56.6%, and 5% strongyle egg reduction were recorded for goats treated with 30, 10, and 3 mg/kg of encapsulated bromelain, respectively. In addition, 32.4% and 96.5% strongyle egg reduction was noted for goats treated with 30 mg/kg of plain bromelain and 7.5 mg/kg of albendazole, respectively. In contrast, the percentage egg count for the negative control group increased by 22% (Figure-3).

## Discussion

The current study was geared toward evaluating the *in vitro* and *in vivo* efficacy and toxicity of a bromelain chitosan formulation which can extend the release of the drug. The *in vitro* results demonstrated that albendazole had higher anthelmintic activity than encapsulated bromelain as well as plain bromelain. This is consistent with the findings by Hunduza [12] who also evaluated the anthelmintic activity of bromelain encapsulated in chitosan nanocarriers against *H. contortus* isolated from naturally infected goat and showed that albendazole had the greatest effect on the worms followed by encapsulated bromelain and extracted bromelain.

The observed albendazole and bromelain LC_50s_ are comparable to those obtained by Cheruiyot [30] and Hunduza [12]. The small difference could be related to varying quality and source of bromelain [31]. The anthelmintic mechanism of action of pineapple cysteine proteinases is well documented [32,33]. These enzymes attack protein targets in the cuticle leading to weakening of the cuticle, blistering and rupture, and subsequent release of internal tissues. These eventually lead to the death of the worm. This property is not limited to the enzymes from pineapple and papaya but has been reported in papain homologs from figure and lattices of other plants [32-34].

Similar to the findings of this study, Dutta and Bhattacharyya [35] did not observe any toxicity after oral administration of acute and sub-acute doses of the aqueous extract of *Ananas comosus* (pineapple) crown leaf to rats. According to Taussig *et al*. [18], bromelain has very low toxicity and its LD_50_ has been evaluated to be >10 g/kg in mice, rates, and rabbits. Pavan *et al*. [19] reported that daily administration of bromelain to dogs in an increasing level up to 750 mg/kg showed no toxic effects after 6 months. Corroborating our results, Moss *et al*. [20] did not find any alteration in the histology of heart, kidney, or hematological parameters after administration of 1500 mg/kg bromelain per day to rats. The PCV observed in this study was lower compared to the values (32.5-43.7%) reported by Al-Bulushi *et al*. [36] for Sahrawi goats. Earlier reports for Jabali goats showed PCV values ranging from 37.4% to 43.7% [36]. Despite the small difference observed, the obtained PCV values were within the normal range of the animal [37]. The observation that there were no significant differences between the PCV of treated and control non-treated goats indicates that the administration of encapsulated bromelain does not affect erythrocyte production and physiology. The serum creatinine, urea, ALT, and AST levels were within the normal range of the animal and did not show any significant difference between the treated and the negative control non-treated goats indicating normal functioning kidneys and liver. The range of goat ALT level observed in this study is comparable with the finding by Tibbo *et al*. [38] who indicated that the ALT level of indigenous Arsi-Bale, Central Highland and Long-eared Somali goat breeds ranged from 14.0 to 20.2 U/L. The obtained values for urea were in agreement with the report of Chikwanda and Muchenje [39] and also Kaneko *et al*. [40] who reported urea level ranging from 3.57 to 7.14 mmol/L, which was in the normal range of the animal.

As compared to the findings of this study, Domingues *et al*. [41] reported a lower efficacy for bromelain in sheep infected with *H. contortus*. The disparity between the anthelmintic efficacy reported by Domingues *et al*. [41] and the findings of this study can be attributed to the differences in the administered dosages and to the fact that they used *H. contortus* infected sheep while the present study dealt with goats. In addition, in this study, the drug was administered for 14 days in contrast to Domingues *et al*. [41] single dose. In the present study, encapsulated bromelain had higher activity than plain bromelain, thus, it is clear that the encapsulation process increased the efficacy of bromelain. A similar result was reported by Hunduza [12]. Ribeiro *et al*. [42] further demonstrated that the encapsulation of extracted plant oils in chitosan nanoparticles leads to an increase in their efficacy against *H. contortus*. The observed increased activity of encapsulated bromelain as compared to plain bromelain can be attributed to the fact that encapsulation of enzymatic drugs stabilizes the protein structure, promotes an active-controlled release and stabilizes the activity presenting greater efficacy [43].

## Conclusion and Recommendations

The current study demonstrates that oral administration of chitosan encapsulated bromelain up to a dosage of 30 mg/kg is not associated with any adverse clinical symptoms, toxicity of the main organs, and mortality in goats. The encapsulation of bromelain in chitosan enhances its anthelmintic properties *in vitro* and *in vivo*, and thus encapsulated bromelain can act as an important substitute to synthetic anthelmintic drugs where anthelmintic resistance has developed. However, further experiments with infected animals are essential to establish the dosage of the encapsulated bromelain to be administered in a single dose for the treatment of goats against gastrointestinal strongyles. In addition, species-specific studies on the efficacy of encapsulated bromelain on strongyles are necessary to evaluate its effectiveness against the entire Strongyloididae family.

## Author’s Contributions

All authors conceived and designed the experiment. SW and JK performed the experiment. NM performed the biochemical assays. SW and JK analyzed and wrote the manuscript. All authors read and approved this manuscript.
